# Efficacy of transcutaneous electrical acupoint stimulation combined with general anesthesia for sedation and postoperative analgesia in minimally invasive lung cancer surgery: A randomized, double‐blind, placebo‐controlled trial

**DOI:** 10.1111/1759-7714.13343

**Published:** 2020-02-16

**Authors:** Jiheng Chen, Yunxiao Zhang, Xiaoxi Li, You Wan, Xinqiang Ji, Wei Wang, Xiaozheng Kang, Wanpu Yan, Zhiyi Fan

**Affiliations:** ^1^ Key Laboratory of Carcinogenesis and Translational Research (Ministry of Education, Beijing), Department of Anesthesiology Peking University Cancer Hospital and Institute Beijing China; ^2^ Neuroscience Research Institute and Department of Neurobiology Peking University and Key Laboratory of Neuroscience of the Ministry of Education and the National Health Commission Beijing China; ^3^ Key Laboratory of Carcinogenesis and Translational Research (Ministry of Education, Beijing), Department of Medical Record Statistics Peking University Cancer Hospital & Institute Beijing China; ^4^ Key Laboratory of Carcinogenesis and Translational Research (Ministry of Education, Beijing), The First Department of Thoracic Surgery Peking University Cancer Hospital and Institute Beijing China

**Keywords:** Analgesia, general anesthesia, sedation, transcutaneous acupoint electrical stimulation, video‐assisted thoracoscopic surgery

## Abstract

**Background:**

Multimodal opioid‐sparing analgesia is a key component of an enhanced recovery pathway after surgery that aims to improve postoperative recovery. Transcutaneous electrical acupoint stimulation (TEAS) is assumed to alleviate pain and anxiety and to modify the autonomic nervous system. This study aimed to determine the efficacy of TEAS for sedation and postoperative analgesia in lung cancer patients undergoing thoracoscopic pulmonary resection.

**Methods:**

A total of 80 patients were randomized into two groups: the TEAS group and the sham TEAS combined with general anesthesia group. Postoperative pain levels at six, 24, 48 hours, and one month after surgery were measured using the visual analogue scale (VAS). Bispectral index (BIS) score during the TEAS prior to anesthetic induction, Observer's Assessment of Alertness/Sedation (OAAS) score, sufentanil consumption during postoperative patient‐controlled intravenous analgesia (PCIA), number of total and effective attempts of PCIA pump use, and incidence of postoperative nausea and vomiting were recorded and analyzed statistically.

**Results:**

Patients in the TEAS group had significantly lower VAS scores at six, 24, and 48 hours after surgery (*P* < 0.01); lower BIS scores at 10, 20, and 30 minutes before induction (*P* < 0.01); lower levels of postoperative sufentanil consumption; lower number of PCIA attempts and effective rates (*P* < 0.01); lower incidences of nausea at 0, six, 24, and 48 hours; and lower incidence of vomiting at 24 hours after surgery (*P* < 0.05). The postoperative OAAS scores were similar between the groups.

**Conclusions:**

TEAS could be a feasible approach for sedation and postoperative analgesia in thoracoscopic pulmonary resection.

## Introduction

The enhanced recovery after surgery (ERAS) pathway has been widely adopted for a broad range of complex surgical procedures to improve the quality of surgical care. As an essential component of most ERAS pathways, multimodal pain management with nonopioid agents or techniques is established to minimize the use of perioperative opioids and to decrease opioid‐related adverse effects (e.g. nausea, vomiting, ileus, pruritus, and respiratory depression) with the goal of improving and expediting patients' recovery after surgery.^1^

Acupuncture, a noninvasive and nonpharmacological adjunctive intervention, has been widely applied for sedative and analgesic purposes.^2^ In addition to the conventional manual acupuncture, electrical acupuncture and transcutaneous electrical acupoint stimulation (TEAS) have also been employed in clinical practice. The acupuncture‐drug compound anesthesia, referred to as transcutaneous electrical acupoint stimulation combined with general anesthesia, has been demonstrated to relieve perioperative anxiety, reduce intraoperative opioid consumption, and improve postoperative analgesic effect.^3^, ^4^ However, its application in modern lung surgery including video‐assisted thoracic surgery (VATS) has rarely been reported. Thus, it is important to evaluate the efficacy of TEAS in VATS pulmonary resection.

To address this question, the present study assessed the effects of TEAS on perioperative analgesia, opioid consumption, recovery, and complications after thoracoscopic pulmonary resection.

## Methods

### Study design

A randomized, double‐blind, placebo‐controlled study with a pre‐ and a post‐test design was conducted in accordance with the Standards for Reporting Interventions in Controlled Trials of Acupuncture recommendations.^5^ All eligible enrolled patients were randomly assigned to the acupuncture‐drug compound general anesthesia group (TEAS group) or the sham TEAS compound general anesthesia group (sham‐TEAS group) in a 1:1 ratio. Computer‐based sample randomization was simultaneously performed at the enrolment of each patient. The allocation code was generated by an independent statistician. None of the anesthesiologists, surgeons, physicians in the post‐anesthesia care unit, or patients were aware of the allocation. This study was registered with the Chinese Clinical Trial Registry (http://www.chictr.org.cn) under the accession number ChiCTR1800016067.

### Participants

Patients undergoing VATS pulmonary resection for early‐stage lung cancer between 5 May 2015 and 1 March 2017 by a single surgeon‐anesthesiologist team (Ke‐Neng Chen and Jiheng Chen) at the First Department of Thoracic Surgery, Peking University Cancer Hospital and Institute were consecutively screened and enrolled. Inclusion criteria were as follows: patients aged 18–64 years, American Society of Anesthesiologists status I or II, patients undergoing lung operation for the first time, and no history of previous acupuncture‐drug compound anesthesia. The exclusion criteria for randomization were as follows: American Society of Anesthesiologists status III, IV, or V; recent use of TEAS or acupuncture; severe comorbidities including pre‐existing coagulopathy, hypertension (systolic pressure ≥ 180 mmHg and/or diastolic pressure ≥ 110 mmHg), cardiovascular disease, and diabetes; history of chronic pain; and history of opioid or alcohol abuse. The present study was approved by the Medical Ethics Committee of Peking University Cancer Hospital (approval No. 2013KT30). Informed consent was obtained from each patient.

### TEAS protocol

All patients were informed by a designated anesthesiologist about the methods of TEAS and anesthesia one day before the surgery. All patients fasted without food and water intake before the surgery. After being transferred to the operating room, all patients received an intravenous drip of 0.04–0.06 mg/kg midazolam. The HANS‐200A TEAS device (Nanjing Jisheng Medical Technology Co., Ltd., Nanjing, Jiangsu, China) was connected to bilateral Hegu (LI 4), Neiguan (PC 6), Houxi (SI 3), and Zhigou (TE 6) acupoints. The frequency of TEAS was 2/100 Hz. Electrodes (50 × 50 mm) were then placed over each acupoint with the pore directly over the marked acupoint. Tegaderms were placed over the electrodes and the participants' skin to fix the electrodes during intervention.

Patients in the TEAS group received electrical stimulation (10–15 mA) for 30 minutes before anesthetic induction, continuous stimulation (30 mA) throughout the surgical procedure, and intermittent stimulations (10–15 mA) for 30 minutes each at six, 24, and 48 hours after the surgery. Patients in the sham‐TEAS group received 4 mA stimulation before and after the surgery in the same manner as mentioned above. No electrical stimulation was applied during the surgery for patients in the sham‐TEAS group. The sensory threshold of electrical stimulation was around 5 mA.

### Anesthesia protocol

After 30 minutes of TEAS or sham‐TEAS, intravenous induction was started with 0.3–0.5 μg/kg sufentanil, 2 mg/kg propofol, and 0.6–1 mg/kg rocuronium bromide. Patients were intubated with a double‐lumen tube at an appropriate depth. Mechanical ventilation was started after the tube position was confirmed by auscultation and fiberoptic bronchoscopy was performed to reconfirm the tube position. During the procedure, 60% oxygen was maintained with a flow rate of 2 L/min, tidal volume of 6–8 mL/kg, and respiratory rate of 12–15 breaths/minute. Propofol and sufentanil were infused continuously, while rocuronium bromide was administered intermittently for anesthetic maintenance. The bispectral index (BIS) was monitored continuously with a multifunctional electrocardiogram monitor during the surgery. Target‐controlled infusion of propofol was performed and the infusion rate of propofol was adjusted based on the BIS (score 40–60). For the target‐controlled infusion of sufentanil, the infusion rate was adjusted based on hemodynamic parameters. At 30 minutes before the end of the procedure, 10 μg sufentanil was administered for analgesia transition. A patient‐controlled intravenous analgesia (PCIA) pump was connected (sufentanil 1.5 μg/mL, priming volume: 0, PCIA dose: 3 mL, background dose: 2 mL/h, interval: 15 minutes, and duration: two days).

### Endpoints

The primary endpoint was postoperative visual analogue scale (VAS) scores at six, 24, and 48 hours after surgery and secondary endpoints were BIS scores during the TEAS prior to anesthetic induction, Observer's Assessment of Alertness/Sedation (OAAS) score, sufentanil consumption during postoperative PCIA, number of total and effective PCIA pump attempts, incidence of postoperative nausea and vomiting (PONV), and VAS scores at one month after the surgery.

### Sample size

We calculated that 37 patients were required for each group to detect a 25% decrease between the groups, assuming a two‐sided type I error (*α*) of 0.05 and a power of 80%. To account for potential loss to the follow‐up and to enable greater statistical power for secondary analyses, the sample size was increased to 80 patients (40 patients per group).

### Statistical analysis

All statistical analyses were carried out using Stata/SE 15.0 software (StataCorp.LP, College Station, TX, USA). Continuous variables were presented as mean ± standard deviation and were compared using unpaired Student's *t*‐test. Dichotomous variables were presented as number of patients and analyzed using χ^2^ or Fisher's exact test as appropriate. The level of significance for all statistical tests was set at 0.05.

## Results

### Patient characteristics

**Figure 1:**
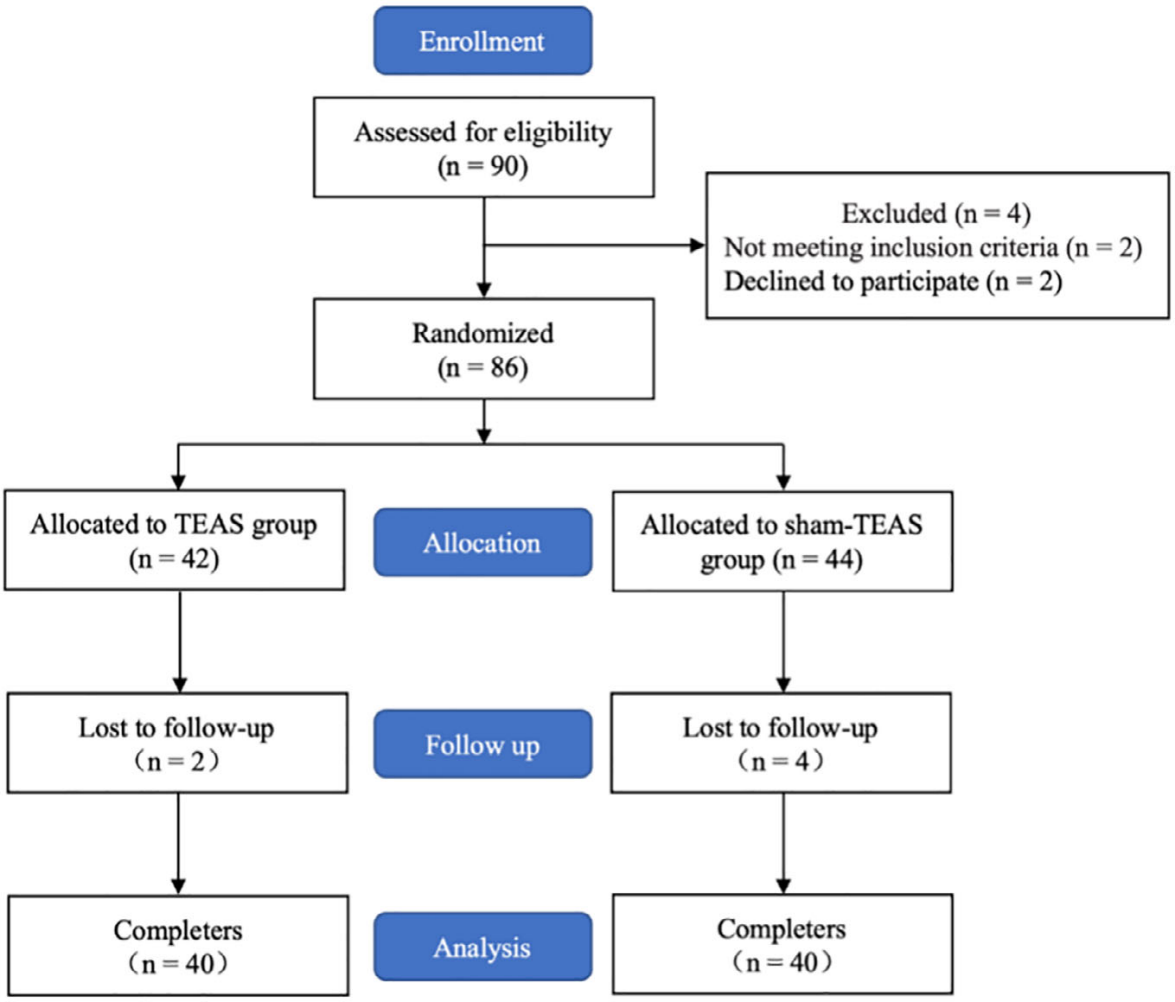
Study flow chart.

Complete datasets were collected for all patients and the data were analyzed (Fig 1). Patient characteristics such as age, gender, body mass index, histology type, type of procedure, duration of surgery, and doses of propofol, sufentanil, and rocuronium bromide did not differ between the groups (Table 1).

**Table 1:** Characteristics of patients enrolled in the study (*n* = 80)

Characteristics	TEAS group (*n* = 40)	Sham‐TEAS group (*n* = 40)	*P*‐value
Age (years)	56.0 ± 3.7	55.8 ± 3.2	0.85
Gender (female/male)	17/23	18/22	0.82
BMI (kg/m^2^)	23.5 ± 0.9	23.4 ± 1.0	0.71
Histology type (ADC/SQCC)	23/17	25/15	0.65
Type of procedure (single/bilobectomy)	19/1	19/1	1.00
Duration of surgery (minutes)	106.28 ± 18.40	111.95 ± 13.60	0.12
Dose of propofol (mg)	969.05 ± 61.37	958.10 ± 61.70	0.43
Dose of sufentanil (mg)	1.28 ± 0.25	1.23 ± 0.21	0.31
Dose of rocuronium bromide (mg)	99.38 ± 22.28	100.75 ± 16.70	0.76

Data are presented as number of patients or mean ± standard deviation.

ADC, adenocarcinoma; BMI, body mass index; SQCC, squamous cell carcinoma; TEAS, transcutaneous electrical acupoint stimulation.

### Primary endpoint

**Figure 2:**
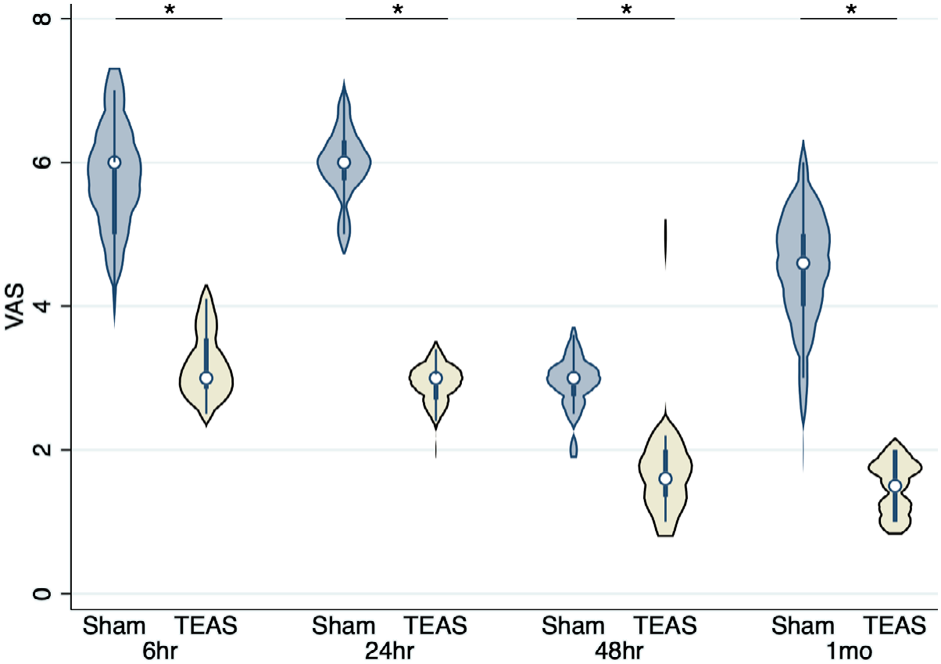
The violin plot of comparison of visual analogue scale (VAS) scores at different times after surgery between the sham‐transcutaneous electrical acupoint stimulation (TEAS) group and the TEAS groups. *P < 0.001.

The changes in VAS are depicted in Fig 2. The mean VAS scores at six, 24, and 48 hours after surgery in the TEAS group were significantly lower than those in the sham‐TEAS group (six hours: 3.21 ± 0.47 vs. 5.78 ± 0.80, respectively, *P* < 0.001; 24 hours: 2.91 ± 0.2 vs. 5.97 ± 0.56, respectively, *P* < 0.001; and 48 hours: 1.69 ± 0.66 vs. 2.92 ± 0.38, respectively, *P* < 0.001).

### Secondary endpoints

**Figure 3:**
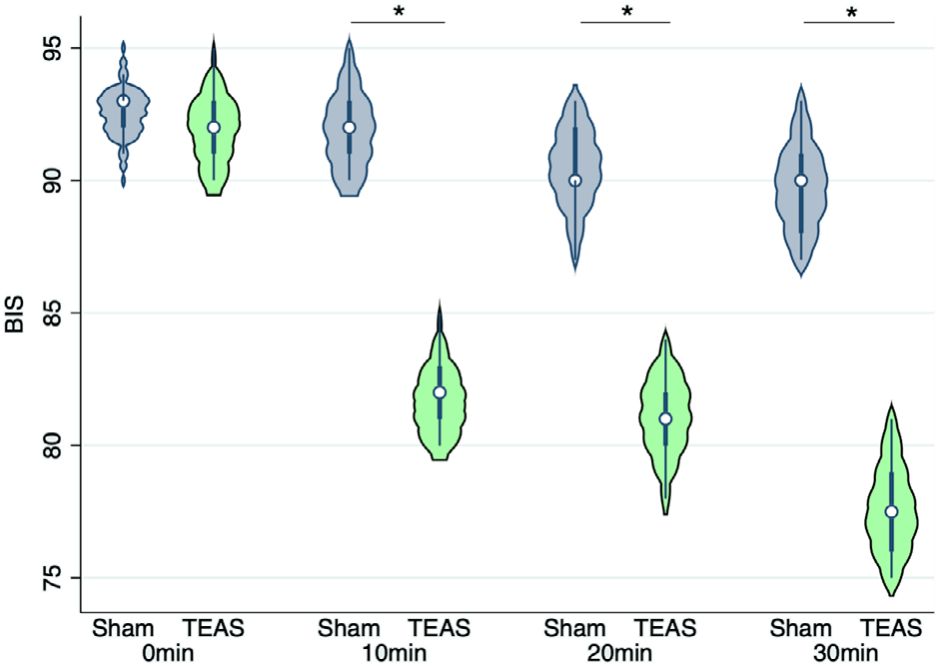
The violin plot of comparison of bispectral index scores at different times before and after the transcutaneous electrical acupoint stimulation (TEAS) between the sham‐TEAS group and the TEAS group. *P < 0.001.

The baseline BIS scores before TEAS were similar between the groups (TEAS vs. sham‐TEAS: 91.95 ± 1.38 vs. 92.53 ± 1.41, *P* = 0.07). The BIS scores at 10, 20, and 30 minutes after preinduction TEAS were significantly lower in the TEAS group than those in the sham‐TEAS group (10 minutes: 81.78 ± 1.37 vs. 91.93 ± 1.46, respectively, *P* < 0.001; 20 minutes: 81.03 ± 1.48 vs. 90.45 ± 1.50, respectively, *P* < 0.001; and 30 minutes: 77.63 ± 1.67 vs. 89.80 ± 1.68, respectively, *P* < 0.001) (Fig 3).

**Figure 4:**
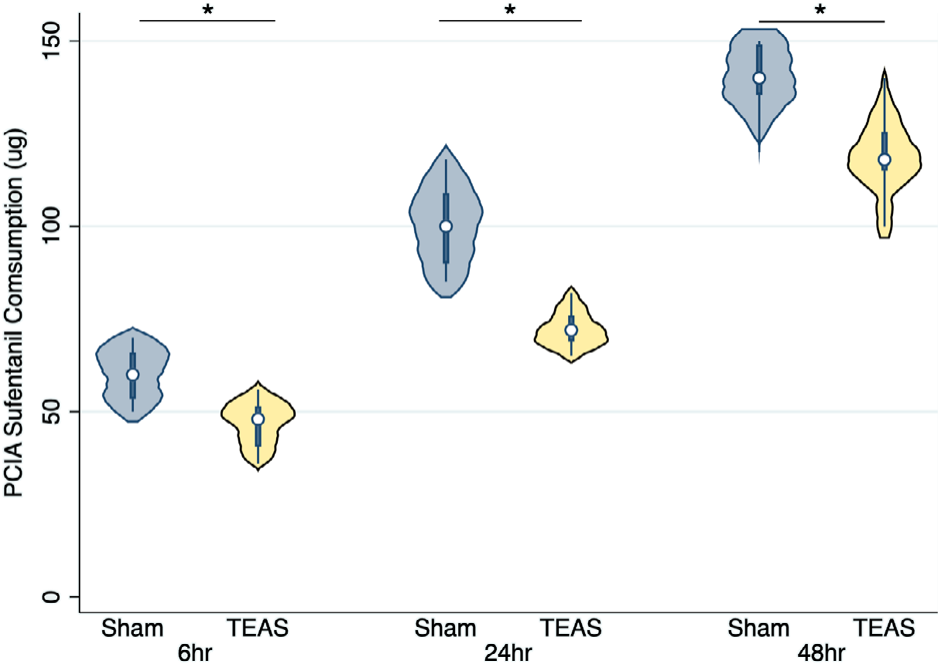
The violin plot of comparison of sufentanil consumption during postoperative patient‐controlled intravenous analgesia at different times after surgery between the sham‐transcutaneous electrical acupoint stimulation (TEAS) group and the TEAS group. *P < 0.001.

The cumulative changes in sufentanil consumption during PCIA after surgery are depicted in Fig 4. The consumption of sufentanil during PCIA at six, 24, and 48 hours in the TEAS group was significantly lower than that in the sham‐TEAS group (six hours: 46.85 ± 5.65 μg vs. 60.08 ± 6.91 μg, respectively, *P* < 0.001; 24 hours: 72.43 ± 4.78 μg vs. 100.62 ± 10.20 μg, respectively, *P* < 0.001; and 48 hours: 118.52 ± 9.77 μg vs. 140.15 ± 7.87 μg, respectively, *P* < 0.001).

**Figure 5:**
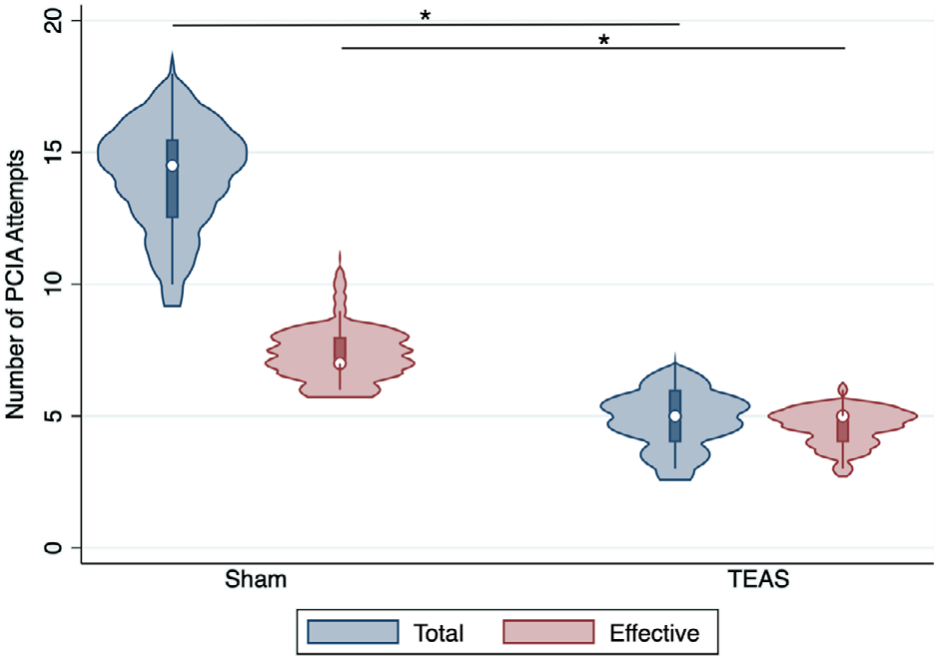
The violin plot of comparison of total and effective patient‐controlled intravenous analgesia attempts after surgery between the sham‐transcutaneous electrical acupoint stimulation (TEAS) group and the TEAS group. *P < 0.001. (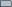) Total, and (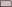) Effective.

The mean number of total and effective PCIA pump attempts in the TEAS group was significantly lower than those in the sham‐TEAS group (mean total attempts: 4.83 ± 1.06 vs. 14.05 ± 2.01, respectively, *P* < 0.001 and mean effective attempts: 4.58 ± 0.93 vs. 7.48 ± 1.18, respectively, *P* < 0.001) (Fig 5).

**Figure 6:**
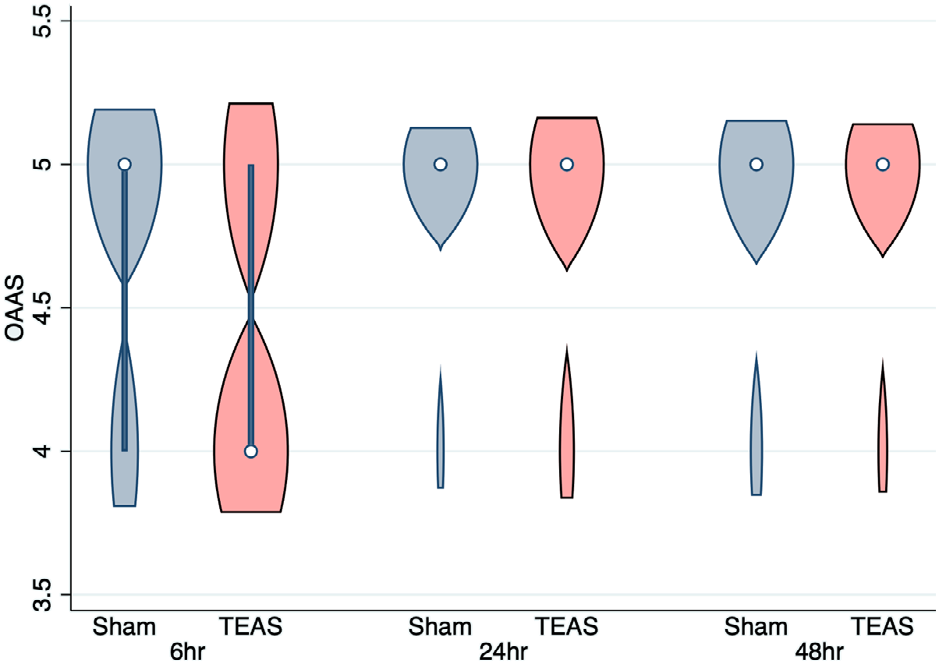
The violin plot of comparison of Observer's Assessment of Alertness/Sedation scores at different times after transcutaneous electrical acupoint stimulation (TEAS) between the sham‐TEAS group and the TEAS groups.

The OAAS scores were similar between the groups at six, 24, and 48 hours after surgery (Fig 6). The VAS scores one month after surgery were significantly lower in the TEAS group than those in the sham‐TEAS group (1.49 ± 0.41 vs. 4.48 ± 0.86, respectively, *P* < 0.001) (Fig 2).

We compared analgesia‐related adverse effects of nausea and vomiting between the groups. Significantly lower incidences of PONV were observed in the TEAS group compared to the sham‐TEAS group at 0, six, 24, and 48 hours after surgery, except in terms of vomiting at 48 hours after surgery (Table 2).

**Table 2:** Analgesic outcomes in the TEAS and the sham‐TEAS groups

Analgesic outcome	TEAS group (*n* = 40)	Sham‐TEAS group (*n* = 40)	*P*‐value
Nausea			
0 hours after surgery	0 (0.0%)	17 (42.5%)	<0.001
6 hours after surgery	6 (15.0%)	27 (67.5%)	<0.001
24 hours after surgery	5 (12.5%)	22 (55.0%)	<0.001
48 hours after surgery	0 (0.0%)	14 (35.0%)	<0.001
Vomiting			
0 hours after surgery	0 (0.0%)	5 (12.5%)	0.03
6 hours after surgery	6 (15.0%)	14 (35.0%)	0.04
24 hours after surgery	1 (2.5%)	11 (27.5%)	<0.001
48 hours after surgery	0 (0.0%)	0 (0.0%)	NS

Data are presented as numbers (percentages).

NS, not significant; TEAS, transcutaneous electrical acupoint stimulation.

## Discussion

To the best of our knowledge, the present study is the first to examine the dynamic impact of TEAS use on postoperative pain and analgesia‐related outcomes in patients undergoing minimally invasive major lung surgery. Preoperative TEAS use was found to be associated with a statistically significant decrease in pain scores in the early and late postoperative periods and a significant decrease in opioid consumption. A lower incidence of PONV was also observed in the early postoperative period in TEAS users.

The acceptance of TEAS use combined with general anesthesia is increasing due to improvement in perioperative outcomes in diverse major surgeries. There is accumulating evidence indicating that TEAS may decrease the stress response during extubation, enhance the anesthetic effect, improve the quality of postoperative recovery, yield a cardioprotective effect, and reduce opioid consumption and related PONV risks.^3^, ^6^, ^7^, ^8^, ^9^, ^10^

We performed a randomized, double‐blind, and placebo‐controlled trial to reduce the probability of bias. We believe that our study was adequately powered to analyze the primary endpoint (difference in the intensity of postoperative pain between the groups compared using the paired *t*‐test), as sample size estimation indicated that 37 patients were required in each group to detect a mean difference of 25% for a study with a type I error of 5% and a type II error of 20%. A 10 mm change in the VAS scores for pain (or a 1‐point difference in the numerical rating scores) has previously been found to signify a clinically important difference for acute postoperative pain.^11^

Lung surgery guidelines strongly recommend opioid‐sparing analgesia and nausea and vomiting control to improve the quality of ERAS.^12^ There is a dose‐response relationship between opioid use and adverse effects.^13^ Our data showed that TEAS reduced the postoperative sufentanil consumption by almost 16%, suggesting that TEAS may exert an analgesic effect. Similarly, the incidences of PONV were dramatically reduced, adding weight to a previous study suggesting analgesic benefit of TEAS for lung cancer patients.^8^ The findings of the present study are significant because prevalence of ERAS is increasing. These findings will lead to an increasing number of modalities for surgery, especially nonpharmacological approaches. Moreover, it is important to know how preoperative TEAS use can impact postoperative outcomes. This knowledge will allow us to study how we can mitigate adverse pain experiences after surgery in the future.

It is well known that the analgesic mechanism of acupuncture is related to endogenous opioid system activation.^14^ Laboratory studies have also shown that continuous electrical stimulation at one frequency could induce tolerance and weaken the analgesic effect.^15^, ^16^ Thus, dilatational wave stimulation has been popularly used in recent years, since it can promote the release of all three endogenous opioid peptides and delay analgesia tolerance under continuous electroacupuncture (EA) or TEAS.^17^ High‐intensity EA produces more extensive and enduring analgesic effect than low‐intensity EA due to the activation of brainstem negative feedback system by Aδ fibers and also by C fibers under noxious stimulation. The human sensory threshold is about 5 mA and to promote the release of endogenous analgesic substances, the intensity of stimulation should be at least 2–3 times the threshold.^18^ The acupoints Hegu and Houxi have a general analgesic effect according to the main and collateral channels theory in Traditional Chinese Medicine. The combined stimulation of the four selected acupoints has a viscera‐balancing effect by regulating the mind and the qi. Therefore, Hegu, Neiguan, Houxi, and Zhigou acupoints were stimulated in the present study for acupuncture‐drug compound anesthesia. The frequency was 2/100 Hz. The intensity was 10–15 mA for pre‐ and postoperative stimulation and 30 mA for intraoperative stimulation. Our results indicated that the combined stimulation of Hegu, Neiguan, Houxi, and Zhigou acupoints during TEAS was effective in preventing PONV.

There are some limitations to the present study. Since the study had a relatively small sample and a single institutional experience, a prospective multicenter study on TEAS is warranted. Moreover, we were able to assess pain and related outcomes only in the first month after the surgery. A study that follows patients over a period of a few months may yield a more accurate profile of postoperative pain trajectories in this population.

In conclusion, the current study demonstrated an association between TEAS use and decreased postoperative pain, decreased use of analgesics, and decreased PONV risk in patients undergoing minimally invasive lung cancer surgery. We recommend this noninvasive and nonpharmacological modality for perioperative analgesia.

## Disclosure

No authors report any conflict of interest.

## References

[1] Wick EC , Grant MC , Wu CL . Postoperative multimodal analgesia pain management with nonopioid analgesics and techniques: A review. JAMA Surg 2017; 152 (7): 691–7. 28564673 10.1001/jamasurg.2017.0898

[2] Wu MS , Chen KH , Chen IF *et al* The efficacy of acupuncture in post‐operative pain management: A systematic review and meta‐analysis. PLOS One 2016; 11 (3): e0150367. 26959661 10.1371/journal.pone.0150367 PMC4784927

[3] Zhang Q , Gao Z , Wang H *et al* The effect of pre‐treatment with transcutaneous electrical acupoint stimulation on the quality of recovery after ambulatory breast surgery: A prospective, randomised controlled trial. Anaesthesia 2014; 69 (8): 832–9. 24865978 10.1111/anae.12639

[4] Wang H , Xie Y , Zhang Q *et al* Transcutaneous electric acupoint stimulation reduces intra‐operative remifentanil consumption and alleviates postoperative side‐effects in patients undergoing sinusotomy: A prospective, randomized, placebo‐controlled trial. Br J Anaesth 2014; 112 (6): 1075–82. 24576720 10.1093/bja/aeu001

[5] MacPherson H , Altman DG , Hammerschlag R *et al* Revised STandards for reporting interventions in clinical trials of acupuncture (STRICTA): Extending the CONSORT statement. PLOS Med 2010; 7 (6): e1000261. 20543992 10.1371/journal.pmed.1000261 PMC2882429

[6] Wong RH , Lee TW , Sihoe AD *et al* Analgesic effect of electroacupuncture in postthoracotomy pain: A prospective randomized trial. Ann Thorac Surg 2006; 81 (6): 2031–6. 16731125 10.1016/j.athoracsur.2005.12.064

[7] Wang XQ , Yu JL , Du ZY , Xu R , Jiang CC , Gao X . Electroacupoint stimulation for postoperative nausea and vomiting in patients undergoing supratentorial craniotomy. J Neurosurg Anesthesiol 2010; 22 (2): 128–31. 20308818 10.1097/ANA.0b013e3181c9fbde

[8] Huang S , Peng WP , Tian X *et al* Effects of transcutaneous electrical acupoint stimulation at different frequencies on perioperative anesthetic dosage, recovery, complications, and prognosis in video‐assisted thoracic surgical lobectomy: A randomized, double‐blinded, placebo‐controlled trial. J Anesth 2017; 31 (1): 58–65. 26350110 10.1007/s00540-015-2057-1

[9] Bai WY , Yang YC , Teng XF , Wan YX , Wei W , Zhu JC . Effects of transcutaneous electrical acupoint stimulation on the stress response during extubation after general anesthesia in elderly patients undergoing elective supratentorial craniotomy: A prospective randomized controlled trial. J Neurosurg Anesthesiol 2018; 30 (4): 337–46. 29076978 10.1097/ANA.0000000000000460

[10] Li H , Wu C , Yan C *et al* Cardioprotective effect of transcutaneous electrical acupuncture point stimulation on perioperative elderly patients with coronary heart disease: A prospective, randomized, controlled clinical trial. Clin Interv Aging 2019; 14: 1607–14. 31564843 10.2147/CIA.S210751 PMC6735632

[11] Myles PS , Myles DB , Galagher W *et al* Measuring acute postoperative pain using the visual analog scale: The minimal clinically important difference and patient acceptable symptom state. Br J Anaesth 2017; 118 (3): 424–9. 28186223 10.1093/bja/aew466

[12] Batchelor TJ , Rasburn NJ , Abdelnour‐Berchtold E *et al* Guidelines for enhanced recovery after lung surgery: Recommendations of the Enhanced Recovery After Surgery (ERAS®) Society and the European Society of Thoracic Surgeons (ESTS). Eur J Cardiothorac Surg 2018; 55 (1): 91–115. 10.1093/ejcts/ezy301 30304509

[13] McQuay H . Opioids in pain management. Lancet 1999; 353 (9171): 2229–32. 10393001 10.1016/S0140-6736(99)03528-X

[14] Tian JH, Zhang W, Fang Y, Xu W, Grandy DK, Han JS. Endogenous or phanin FQ: evidence for a role in the modulation of electro acupuncture analgesia and the development of tolerance to analgesia produced by morphine and electro acupuncture. *Br J Pharmacol* 1998; **124** (1): 21–26. 10.1038/sj.bjp.0701788 PMC1565350 9630338

[15] DeSantana JM , da Silva LFS , Sluka KA . Cholecystokinin receptors mediate tolerance to the analgesic effect of TENS in arthritic rats. Pain 2010; 148 (1): 84–93. 19944533 10.1016/j.pain.2009.10.011 PMC3954516

[16] Chandran P , Sluka KA . Development of opioid tolerance with repeated transcutaneous electrical nerve stimulation administration. Pain 2003; 102 (1–2): 195–201. 12620611 10.1016/s0304-3959(02)00381-0

[17] DeSantana JM , Santana‐Filho VJ , Sluka KA . Modulation between high‐and low‐frequency transcutaneous electric nerve stimulation delays the development of analgesic tolerance in arthritic rats. Arch Phys Med Rehabil 2008; 89 (4): 754–60. 18374009 10.1016/j.apmr.2007.11.027 PMC2744433

[18] Han JS . Acupuncture: Neuropeptide release produced by electrical stimulation of different frequencies. Trends Neurosci 2003; 26 (1): 17–22. 12495858 10.1016/s0166-2236(02)00006-1

